# Age effect on inter-pulse interval selection for ECAP measurement

**DOI:** 10.3389/fnins.2025.1647513

**Published:** 2025-08-29

**Authors:** Han Wu, Xue-ying Yang, Chao-qun Liang, Jian-ming Yang

**Affiliations:** ^1^Department of Otolaryngology Head and Neck Surgery, Second Hospital of Anhui Medical University, Hefei, China; ^2^Department of Otolaryngology Head and Neck Surgery, First Affiliated Hospital of Soochow University, Suzhou, China

**Keywords:** cochlear implant, electrically evoked compound action potential, inter-pulse interval, age, Nurotron

## Abstract

**Introduction:**

Age and duration of deafness significantly affect the auditory nerve (AN) health of cochlear implant (CI) users. In this study, we examine how age and duration of deafness impact Electrically Evoked Compound Action Potential (ECAP) amplitudes when the Inter-Pulse Interval (IPI) parameter in ECAP measurements is varied.

**Methods:**

Our participants were thirty-one Mandarin-speaking CI users with Nurotron/CS-20A (19 males and 12 females, 33 ears, aged 6–66 yrs). We investigated eleven distinct IPI settings, ranging from 290 μs to 590 μs (IPI = 290, 330, 350, 390, 410, 450, 470, 510, 530, 570, 590 μs).

**Results:**

The first result of our study was to find the optimal IPI of the participants. Furthermore, the optimal IPI was positively correlated with both the age of the participants (*r* = 0.6018, *p* = 0.0002) and the duration of deafness (*r* = 0.3479, *p* = 0.0473).

**Discussion:**

Our study sheds light on the pivotal role of age and duration of deafness in ECAP amplitudes and optimizing IPI settings. We recommend that CI users with advanced age and long duration of deafness use longer IPI parameters for ECAP measurements to obtain a more accurate ECAP waveform. These insights hold significant implications for tailoring CI programming and rehabilitation strategies to cater to the distinct needs of individuals across different ages and duration of deafness groups, ultimately enhancing the auditory experiences and outcomes of CI users.

## 1 Introduction

Among Neural Response Measurement (NRM) techniques employed in Forward Masking (FM) methods, the Inter-Pulse Interval (IPI) stands out as a critical parameter. IPI is aimed at utilizing the absolute refractory period (ARP) of the auditory nerve response to eliminate artifacts in NRM. FM uses the “masker” to denote the initial pulse and the “probe” to refer to the subsequent one. A pair of biphasic current pulses are delivered in one of the stimulation conditions with a brief IPI between them. When employing sufficiently short IPIs, the neural reaction to the probe is influenced by the preceding masker’s presence. The recorded data in this case combines the two stimulation artifacts and the neural reaction to the masker. In the masker-plus-probe stimulation condition, the neural reaction to the probe is either absent or diminished (Brown et al., 1990).

The concept of the ARP is another critical element to consider. ARP delineates the time elapsed between the generation of an action potential and the subsequent potential for the neuron to fire another action potential when subjected to adequate stimulation. Over the years, several studies with cochlear implant (CI) users have estimated ARP across various patient populations, shedding light on this essential aspect of neural processing (Morsnowski et al., 2006; Hughes et al., 2012; Wiemes et al., 2016; He et al., 2018; Skidmore et al., 2021). The IPI, a crucial variable in our study, systematically varies between the initial pulse (the masker) and the subsequent one (the probe). As the IPI increases, the AN embarks on a journey of gradual recovery from the refractoriness imposed by the action potential generated by the first pulse. This journey translates into decreased thresholds and amplified neural responses, particularly noticeable at longer IPIs (Skidmore et al., 2022).

The ARP directly reflects the health and functional integrity of auditory nerve fibers. The ARP tends to be relatively short, allowing for rapid neural responses to electrical stimulation, in individuals with healthy auditory nerve fibers. However, the ARP may be lengthened in cases where the auditory nerve fibers are damaged or have reduced functionality. Importantly, the ARP is not a fixed parameter and may vary between different CI users. Neural health, which can be influenced by factors such as age and duration of deafness, directly affects ARP. Therefore, different groups of CI users may have different ARP values, requiring individualized adjustments of the IPI for optimal neurostimulation results.

Based on the above, we propose the hypothesis that each CI user will have a different optimal IPI for obtaining the Electrically Evoked Compound Action Potential (ECAP) in NRM. The duration of this interval profoundly impacts ECAP amplitude, consequently influencing the ability of clinical practitioners to identify ECAP waveforms. When IPI is inappropriately selected, leading to ECAP amplitudes that are too small to ascertain if they have been elicitated, it is common practice to increase the stimulation level to observe the ECAP threshold. However, this approach comes with two potential drawbacks.

Firstly, CI users may experience discomfort if the stimulation level is increased too much. This discomfort can include physical sensations such as pain or a tingling sensation, as well as perceptual distortions in the experience of hearing. In addition, excessive stimulation levels can potentially damage the delicate neural fabric within the cochlea.

Secondly, it compromises the accuracy of ECAP threshold determination. Previous studies have already established that ECAP thresholds can serve as a predictor for the T- and C-level, which is particularly valuable for CI users especially young children who cannot provide subjective feedback. There were great variations of correlation coefficients across different studies, with moderate to strong correlations between ECAP thresholds and T- or C-levels, with correlation coefficient variations ranging between *r* = 0.5 to 0.9 (Di Nardo et al., 2003; Smoorenburg et al., 2002; Cullington, 2000; Polak et al., 2005; Potts et al., 2007; Allam and Eldegwi, 2019; Chao et al., 2023).

Studies have shown that aging causes AN degeneration in human CI users. Makary et al. (2011) assessed the primary loss of spiral ganglion cells (SGCs) in human ears and found that ganglion cell counts declined at a mean rate of 100 cells per year of life. Wu et al. (2019) used autopsy material from 20 subjects aged 0 to 89 yrs. and the results suggest that a large number of auditory neurons in the aging ear are disconnected from their hair cell targets. As CI users age, the auditory system undergoes natural changes. These changes can lead to reduced neural responsiveness.

In addition to age, the duration of deafness before cochlear implantation is another crucial factor. The impact of auditory deprivation and duration of deafness on postoperative performance has to be taken into account while counseling suitable candidates for CI. Longer duration of deafness seems to lead to worse CI performance (Zeh and Baumann, 2015; Bernhard et al., 2021). Studies revealed that the duration of deafness can lead to modifications of cortical and subcortical brain regions in patients with asymmetric as well as bilateral hearing loss (Anderson et al., 2017; Speck et al., 2020; Simon et al., 2020). Prolonged periods of hearing loss can lead to neural deafferentation and the loss of auditory nerve fibers due to disuse. Longer duration of deafness has been associated with reduced neural survival rates, leading to compromised auditory nerve health.

Auditory deprivation in prelingual deafness may affect the development of auditory neural pathways, which need stimulation to mature. The maturation of the central auditory pathways and the development of speech and language skills are affected by this deprivation. In prelingual children, the lack of auditory input during the primary stages of development may alter the normal maturation of the central auditory system. In postlingual deafness, the neural pathways are already formed and there is an auditory memory (Carvalho et al., 2020). During the first 6 years of life, a period of high neural plasticity was reached with CI stimulation, close to that of a normal listener (Harrison et al., 2005). Alvarez et al. (2010) found there was a difference in the C-levels measured by the audiologist that were greater in prelingual than in postlingual patients. These studies suggest differences in the auditory nerve between prelingual and postlingual patients.

Over the months or even years following cochlear implantation, activation of the associative auditory cortex gradually increases for stimuli containing speech information but remains stable for noise. A central learning process therefore occurs in implanted patients enabling them to distinguish information with linguistic content from other information (Giraud et al., 2001). Studies have found that auditory nerve degeneration can be prevented by chronic electrical stimulation (Miller et al., 2002).

It is reasonable to hypothesize that these factors may also influence the selection of the optimal IPI in NRM, given the established impact of age, duration of deafness, prelingual or postlingual deafness, and CI experience on AN health. Specifically, it is conceivable that CI users with advanced age, longer duration of deafness, prelingual deafness, and shorter CI experience may require longer IPIs to achieve robust ECAP responses due to the potential compromise in AN health.

This study tested the hypothesis that each CI user has a unique optimal IPI. The optimal IPI increases with age, duration of deafness, and CI experience. In addition, prelingual deafness patients have a longer optimal IPI. By testing these hypotheses, we aim to illuminate the complex relationships between CI users and optimal IPI selection in NRM. In addition, by finding more accurate ECAP thresholds for different CI users, we hope to be able to better predict T- and C-levels.

## 2 Materials and methods

### 2.1 Participants

Thirty-one Mandarin-speaking CI users with Nurotron/CS-20A (19 males and 12 females, 33 ears) participated in the study. The mean age at testing was 29.2 ± 19.5 yrs. (range = 6.0–66.0 yrs), the mean duration of deafness was 5.7 ± 5.4 yrs. (range = 0.5–20.0 yrs), and the mean CI experience was 0.2 ± 0.2 yrs. (range = 0.1–0.8 yrs). CI subject demographic information is shown in Table 1. All participants underwent Cone Beam Computed Tomography (CBCT) after the CI surgery to ensure proper implantation.

**Table 1:** Subject demographic information for the experiment.

Subject	Gender	Age at test (yrs)	Dur deaf (yrs)	Etiology	CL exp.(yrs)	CI ear	Pre- or Post-lingual deaf
S1	M	33	14.0	Unknown	0.3	L	Post
S2	M	7	7.0	Congenital	0.8	R	Pre
S3	F	6	6.0	Congenital	0.7	L	Pre
S4	M	43	6.0	Infectivity	0.5	L	Post
S5	M	24	3.0	Drug-induced	0.3	L	Post
S6	M	41	15.0	Unknown	0.1	R	Pre
S7	M	36	0.5	Unknown	0.1	L	Post
S8	M	8	5.0	LVAS	0.1	L	Pre
S9	M	66	10.0	Sudden	0.1	L	Post
S10	F	10	9.0	Congenital	0.1	L	Pre
S11	M	19	0.5	Unknown	0.8	R	Post
S12	M	34	1.0	Unknown	0.2	L	Post
S13	M	49	5.5	Sudden	0.2	R	Post
S13	M	49	7.5	Sudden	0.2	L	Post
S14	F	30	5.0	Unknown	0.1	L	Post
S15	M	6	2.0	Congenital	0.1	R	Pre
S16	F	27	5.5	Sudden	0.1	R	Post
S17	F	13	0.8	Unknown	0.1	L	Post
S18	F	38	20.0	Unknown	0.2	R	Post
S19	M	7	2.0	Congenital	0.2	R	Pre
S20	M	38	1.0	Unknown	0.1	R	Post
S21	M	7	1.3	LVAS	0.2	L	Post
S22	F	46	5.5	Sudden	0.1	R	Post
S23	M	61	13.0	Sudden	0.3	L	Post
S24	M	56	0.5	Unknown	0.1	L	Post
S25	F	8	3.5	Congenital	0.3	L	Pre
S25	F	8	3.0	Congenital	0.3	R	Pre
S26	F	27	6.0	Unknown	0.1	R	Post
S27	F	29	0.5	Drug-induced	0.2	L	Post
S28	M	44	5.0	Otitis media	0.3	R	Post
S29	F	17	1.5	LVAS	0.1	R	Post
S30	F	56	20.0	Drug-induced	0.1	R	Post
S31	M	20	1.0	Drug-induced	0.3	R	Post

F, female; M, male; Dur deaf, duration of deafness; CI exp., CI experience; L, left; R, right; LVAS, Large Vestibular Aqueduct Syndrome; Pre, prelingual deaf; Post, postlingual deaf.

### 2.2 ECAP measurement

#### 2.2.1 Neural response measurement (NRM) configurable platform

In the present study, we used the algorithm based on the NRM configurable platform, which is newly developed by Nurotron Biotechnology Inc., and is specifically utilized in CS-20A cochlear implants (Yang et al., 2024). The platform allows to manipulation of the parameters of the NRM system for conducting electrophysiology research. The stimulating controller regulates the stimulating circuit and the stimulating switch bank SE1 to SE24, which are connected to 24 corresponding electrodes. The data and commands from the external device determine the settings of the stimulating waveform’s amplitude, duration, and gap in the stimulating circuit. The switch bank can also control parameters such as the IPI, stimulating electrode (SE), and stimulation rate (SR). These parameters can be adjusted using the NRM test module embedded in the NuroSound fitting software. The measurement system comprises the recording switch bank SR1 to SR24, amplifier (AMP), auto-zero offset cancellation circuit, analog-to-digital converter (ADC), and accumulative register. The recording switch bank serves as an independent switch bank used to connect 24 electrodes in Nurotron’s CI. As a result, both the stimulating and recording electrodes can be selected without any restrictions. The amplifier includes a bandpass filter designed to suppress undesired noise and offers four different selectable gains to select (32 dB, 44 dB, 52 dB, and 64 dB). To continually cancel the system offset, the auto-zero offset cancellation circuit is employed to keep canceling the system offset. When SA is enabled, the cancellation circuit becomes active, causing the output of the AMP to remain at 0. Conversely, when SA is disabled, the circuit is deactivated, allowing the measurement system to function properly. For the conversion of the amplified signal to digital data, a 12-bit successive approximation (SAR) ADC with a maximum sample rate of 250 KHz is utilized. Finally, the output data from the ADC is stored in the accumulative register. Once the system measurement reaches the set accumulative times (AT), all the data is transmitted by the back telemetry circuit.

#### 2.2.2 Parameter settings

Pulses Gap = 10 μs, Pulse Width = 50 μs, Gain = 44 dB, Stimulation rate = 33.3 Hz, Iteration (N) = 10. The system access delay for measurements (Ts) and the access delay for recording electrodes (Te) were set at 5 μs and 25 μs, respectively. Stimulation levels = 80 CU, 100 CU, 120 CU, 140 CU. The five test electrodes were spaced across the array, at every sixth electrode (1, 7, 13, 19, 24). The recording electrode was located adjacent to the test electrode (2, 8, 14, 20, 23). Eleven sets of IPI parameters were included, ranging from 290 μs to 590 μs (IPI = 290, 330, 350, 390, 410, 450, 470, 510, 530, 570, 590 μs). As a result, there were 220 ECAPs for each subject.

#### 2.2.3 Indicators

**Figure 1:**
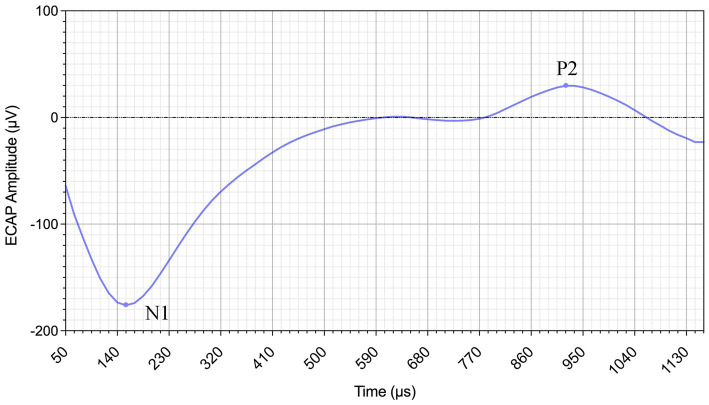
Example ECAP waveform from Subject 1 recorded at electrode 1 with a stimulation level of 140 CU. The N1 and P2 peaks are indicated.

ECAP amplitudes were defined as the voltage difference between the positive peak (P2) and negative peak (N1). The N1 occurs approximately 0.2–0.4 ms after stimulation onset, and the P2 appears around 0.6–0.8 ms (Cullington, 2000). These peaks were recorded as automatically detected by Nurosound but modified by an experienced observer if necessary. Representative ECAP waveform from Subject 1 at electrode 1 with a stimulation level of 140 current units (CU) is shown in Figure 1.

The optimal IPI was determined by calculating the highest mean ECAP amplitude across the five test electrodes by four stimulus intensities. Each subject obtained their optimal IPI through experimentation.

#### 2.3 Statistical methods

Collected data were subjected to normality tests to assess data distribution. Depending on the distribution and study design, independent-samples t-tests were performed to compare group means. In addition, correlation and linear regression analyses were conducted. Although multiple correlation tests were conducted (e.g., between optimal IPI and demographic factors such as age, duration of deafness, and CI experience), these were limited in number and based on prior hypotheses. Therefore, we did not apply corrections such as Bonferroni adjustment, following recommendations for exploratory analyses in small datasets. The statistical analyses were carried out using GraphPad Prism 8.0.2 and SPSS 25.0. A *p*-value of less than 0.05 was considered statistically significant.

## 3 Results and discussion

### 3.1 Selection of optimal IPI

**Figure 2:**
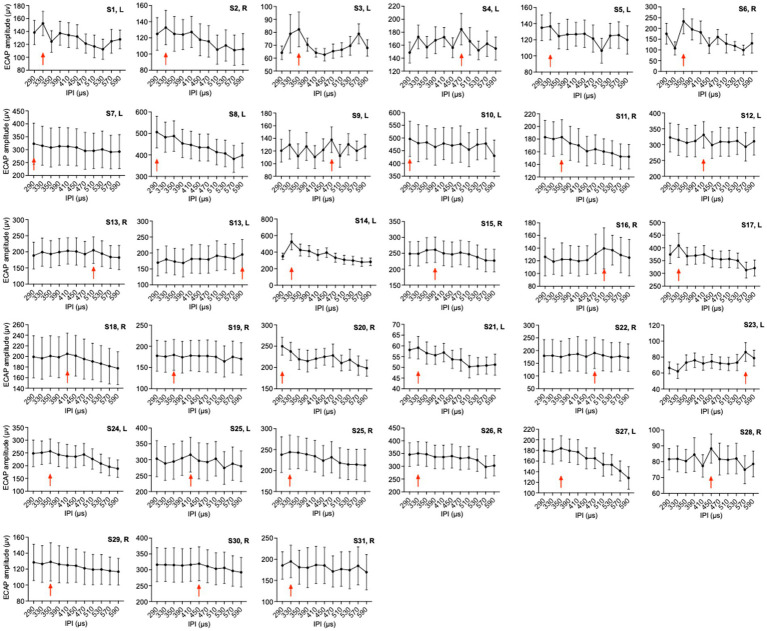
Mean ECAP amplitudes of 33 ears of 11 IPIs. The red arrows indicate the group with the highest mean amplitude. The error bars show the standard deviation.

Figure 2 shows the 33 ears’ means ECAP amplitudes and standard errors at each IPI. Comparisons of ECAP amplitude magnitudes were conducted to assess the influence of various IPI parameters on NRM. The optimal IPI was defined as the point where the ECAP amplitude reached its maximum. The results indicate that each ear has a unique optimal IPI, and variations in IPI also lead to different changes in ECAP amplitudes for each ear.

Although this study determined optimal IPI based on the presence or absence of measurable ECAPs across different pulse intervals, we did not collect full amplitude growth functions (AGFs) for each IPI parameter. AGF slope and threshold measures have been shown to reflect neural health—such as spiral ganglion neuron density—and to vary with interpulse gap settings. For example, increasing the interphase gap (IPG) has been associated with increases in both ECAP peak amplitude and AGF slope in human CI users, with inter-subject variability across electrode sites (Schvartz-Leyzac and Pfingst, 2016; Imsiecke et al., 2021; Gärtner et al., 2021). Moreover, ECAP AGF slopes have been negatively correlated with duration of hearing loss and age at implantation, implying their utility as indicators of auditory nerve condition (Gärtner et al., 2021). Although we did not measure AGFs in the present work, future studies could incorporate AGF-based threshold detection to more sensitively assess how optimal IPI affects neural recruitment and excitability.

It is important to note that the stimulation rates used during ECAP recordings in this study are much lower than the clinical stimulation rates typically used in cochlear implant processors (e.g., 500–3,600 pulses per second). Lower-rate ECAP measurements are necessary to isolate neural responses and minimize overlapping artifacts. However, these reduced rates may not fully replicate the temporal dynamics of everyday CI stimulation. This difference could influence the observed optimal IPI, as neural refractoriness and temporal integration mechanisms behave differently at high stimulation rates (Wilson et al., 1993; Litvak et al., 2007). Therefore, while the optimal IPI identified through ECAP may reflect basic neural excitability, translating this parameter into clinical fitting should consider the temporal properties of real-world CI use.

IPI may influence cochlear implant users’ auditory performance by modulating neural excitability and temporal coding. Shorter IPIs can enhance neural firing synchrony and temporal resolution, which supports better tracking of speech cues, whereas excessively short IPIs may lead to increased neural adaptation and channel interaction that degrade spectral and temporal fidelity (Goldwyn et al., 2012). Human ECAP-derived measures—such as refractory recovery, amplitude-growth-function slope, and interphase-gap effects—have been correlated with inferred neural health and, in some cases, with speech perception outcomes (Schvartz-Leyzac et al., 2020). Although results across studies vary, these findings collectively suggest that optimal IPIs, as derived from ECAP tests, may provide electrophysiological insights into behavioral performance. Future research should directly examine the relationship between ECAP-based IPIs and speech perception under clinically relevant stimulation parameters.

### 3.2 Correlation between subject demographic information and optimal IPI

#### 3.2.1 Age and optimal IPI

**Figure 3:**
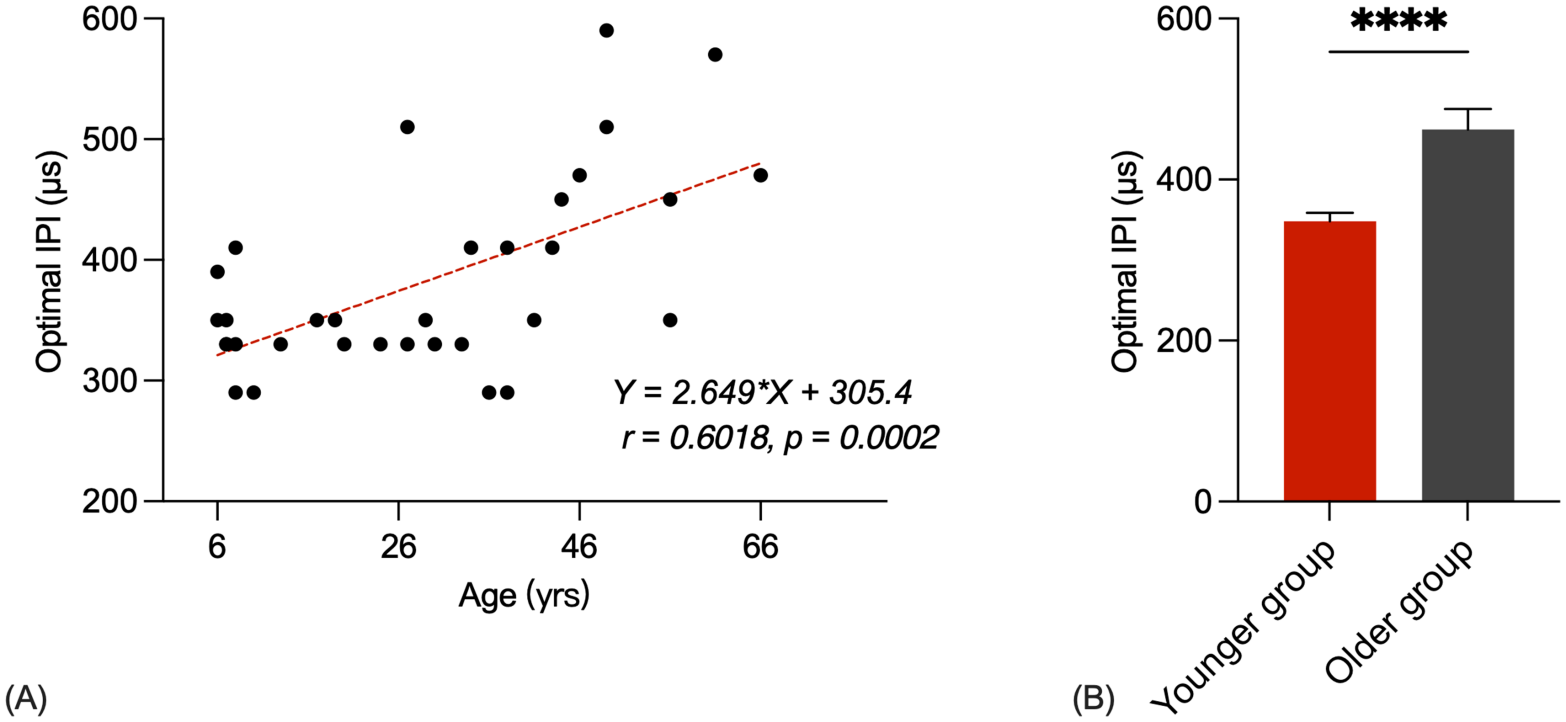
**(A)** Scatter plots for optimal IPIs of ages. The correlation coefficient and *p*-value are shown in the panel. The red line represents the linear fit to the data. **(B)** Younger group and older group optimal IPIs bar chart (*p* < 0.05). The error bars show the standard error.

Figure 3A shows the optimal IPI plotted as a function of the participant’s age at testing. The solid line represents the result of linear regression. Pearson product–moment correlation coefficient and *p*-value are also shown in the panel (*r* = 0.6018, *p* = 0.0002). The optimal IPI generally increases as the age at testing increases. These statistical results suggested a strong, positive correlation between age at testing and the optimal IPI.

Figure 3B shows bar charts of the younger group and older group optimal IPIs. The first group consisted of 22 younger listeners (mean = 19.7 years, range = 6–38 years) and the second group consisted of 9 older listeners (mean = 51.1 years, range = 41–66 years). A Student’s *t*-test showed that the younger group’s optimal IPIs were significantly shorter than those of the older group [*t*(31) = 4.931, *p* < 0.0001]. The average optimal IPI for the younger group was recorded as 348.3 μs, while the older group exhibited an average optimal IPI of 426.0 μs.

Also in agreement with the results of the present study, multiple studies have reported CI users with advanced age and long duration of deafness have poorer AN health. They used different methods to verify AN survival results for various patient populations.

In human CI users, the ARP and the RRP can be estimated based on the ECAP refractory recovery function (RRF). The ECAP RRF is typically measured with two biphasic, charge-balanced, electrical pulses using a modified template subtraction method (Miller et al., 2002). The present study used the classic two-pulse forward-masking paradigm (Brown et al., 1990). In other studies, neural adaptation of the auditory nerve can be evaluated by measuring ECAP amplitudes in response to individual pulses in a constant-amplitude pulse train using a modified forward-masking paradigm (Brown et al., 1990; Wilson et al., 1997; Rubinstein et al., 1999; Miller et al., 2002; Hughes et al., 2014; McKay et al., 2013; He et al., 2016). In this paradigm, the masker-probe interval (MPI) is adjusted to correspond to the period of the pulse rate minus the duration of one biphasic pulse. With this increased MPI duration, coupled with the constant level pulses, some neural response is expected to be evoked by each successive pulse due to partial recovery from refractoriness. In an iterative process, the number of pulses comprising the masker is increased by one, with the final pulse in the pulse train always designated as the probe.

Several studies have investigated refractory properties of the auditory nerve in some special patient populations, including children with auditory neuropathy spectrum disorder (ANSD) (Fulmer et al., 2011), elderly CI users (Lee et al., 2012), and children with cochlea nerve deficiency (CND) (He et al., 2018). Results of these studies showed that children with ANSD had similar refractory recovery time constants compared with children with typical sensorineural hearing loss (SNHL) (Fulmer et al., 2011). Specifically, Lee et al. (2012) compared ECAP refractory recovery rates between younger (age 28–57 years) and older (age 61–89 years) CI users and found no group difference.

On the contrary, He et al. (2018) found out that children with cochlear nerve deficiency (CND) have significantly longer ARPs. Children with CND had significantly higher ECAP thresholds, smaller maximum ECAP amplitudes, flatter slopes of I/O functions, and longer ARPs than children with normal-size cochlear nerves (CNs). This result suggests that poor AN survival results in prolonged ARPs at the population level. In general, larger ECAP amplitude is reported in patient populations with better AN health (He et al., 2020; Luo et al., 2020; Xu et al., 2020).

#### 3.2.2 Duration of deafness and optimal IPI

**Figure 4:**
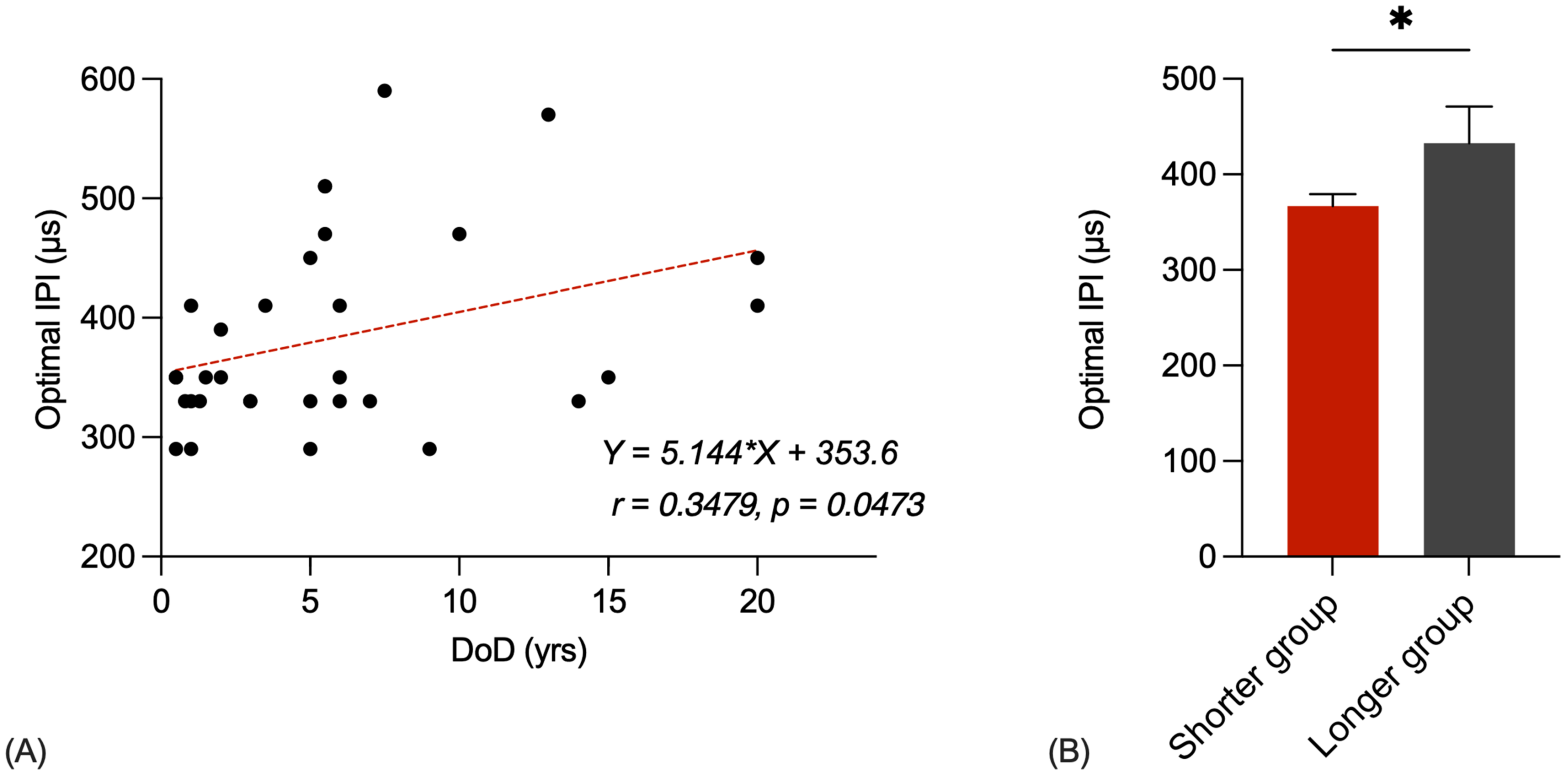
**(A)** Scatter plots for optimal IPIs of durations of deafness. The correlation coefficient and p-value are shown in the panel. The red line represents the linear fit to the data. **(B)** Shorter group and longer group optimal IPIs bar chart (*p* < 0.05). The error bars show the standard error.

Figure 4A shows the optimal IPI plotted as a function of the participant’s duration of deafness at testing. The solid line represents the result of linear regression. Pearson product–moment correlation coefficient and *p*-value are also shown in the panel (*r* = 0.3479, *p* = 0.0473). The optimal IPI generally increases as the duration of deafness at testing increases. These statistical results suggested a strong, positive correlation between the duration of deafness at testing and the optimal IPI.

Figure 4B shows bar charts of the shorter group and longer group optimal IPIs. The first group consisted of 25 listeners who had a shorter duration of deafness (mean = 3.1 years, range = 0.5–7.0 years) and the second group consisted of 8 listeners who had a longer duration of deafness (mean = 13.6 years, range = 7.5–20.0 years). A Student’s t-test showed that the shorter group’s optimal IPIs were significantly shorter than those of the longer group [*t*(31) = 2.132, *p* = 0.0411]. The average optimal IPI for the shorter group was recorded as 366.8 μs, while the longer group exhibited an average optimal IPI of 432.5 μs.

These results underscore the impact of the duration of deafness on the choice of optimal IPI. The observed trend of longer optimal IPIs in individuals with an extended history of deafness implies that the auditory system’s adaptation to electrical stimulation may be influenced by the duration of auditory deprivation. As a consequence, clinicians and researchers should consider the duration of deafness as a key factor when determining the optimal IPI for cochlear implant users. Understanding this relationship can aid in optimizing stimulation parameters for individual patients and potentially enhance their auditory outcomes and overall quality of life. Further research in larger and more diverse cohorts may provide deeper insights into the complex interplay between the duration of deafness, optimal IPI selection, and auditory performance.

These findings suggest that longer auditory deprivation may be associated with delayed neural recovery, reflected by longer optimal IPIs. This is consistent with previous electrophysiological and histological studies that link extended duration of deafness to poorer auditory nerve survival and reduced temporal resolution. For instance, Shepherd and Hardie (2001) showed in animal models that prolonged deafness leads to loss of spiral ganglion neurons, which in turn slows neural response to electrical stimulation. Similarly, Hughes et al. (2000) reported flatter ECAP amplitude growth functions and longer latencies in patients with longer durations of deafness, which may reflect decreased neural synchrony.

However, other human studies have shown inconsistent or weak correlations between deafness duration and ECAP measures. For example, Briaire and Frijns (2005) noted that while duration of deafness may influence some neural response parameters, its effects are often masked by inter-individual variability and electrode-specific factors. Moreover, He et al. (2018) found no significant correlation between ECAP latency and duration of deafness in a cohort of adult CI users.

#### 3.2.3 Pre- or post-lingual deaf and optimal IPI

Our result shows the difference between the prelingual deaf group and the postlingual deaf group optimal IPIs. The first group consisted of 9 listeners who were prelingually deaf and the second group consisted of 24 listeners who were postlingual deaf. A Student’s *t*-test showed that the prelingual deaf group’s optimal IPIs were no significantly shorter than those of the postlingual deaf group [*t*(31) = 1.793, *p* = 0.0829]. The average optimal IPI for the postlingual deaf group was recorded as 343.3 μs, while the postlingual deaf group exhibited an average optimal IPI of 397.5 μs.

Although not statistically significant, the observed trend toward longer optimal IPIs in prelingually deaf individuals may reflect underlying differences in auditory system development and neural encoding efficiency. Prelingual deafness often results in atypical auditory pathway maturation due to early auditory deprivation, which can lead to delayed or degraded neural responses to electrical stimulation. This is consistent with findings by Sharma et al. (2002) and Eggermont and Ponton (2003), who reported prolonged auditory brainstem and cortical response latencies in prelingually deaf children following implantation, indicating altered neural timing.

However, some studies have found minimal or inconsistent differences in ECAP or electrically evoked auditory brainstem response (EABR) measures between pre- and postlingually deaf CI users. For example, Hughes et al. (2000) emphasized that individual variability in neural survival and electrode location may overshadow group-level effects of deafness onset.

The lack of statistical significance in our study may also be attributed to the small sample size, particularly in the prelingual group, and to age differences between the groups. In our sample, prelingual participants were generally younger than postlingual ones, introducing a potential confounding effect, as neural responsiveness and recovery properties may vary with age. Future studies with better age-matched groups and larger samples are necessary to determine whether pre- versus postlingual deafness has a measurable impact on optimal IPI.

#### 3.2.4 CI experience and optimal IPI

Our result shows the optimal IPI and the participant’s CI experience at testing. Correlation analysis showed that there was no significant correlation between optimal IPI and CI experience (*r* = −0.0540, *p* = 0.7644).

The absence of significant differences in our experiment may be attributed to the relatively limited variation in CI experience within our sample of 33 ears. The mean CI experience was 0.2 ± 0.2 years, indicating that most participants had only recently received their implants. Given previous evidence that prolonged CI use may lead to changes in neural responsiveness and auditory pathway plasticity, our findings should be interpreted with caution. For example, Gordon et al. (2006) and Tillein et al. (2010) reported improved neural synchrony and auditory cortical activation over time in children and animals following chronic CI stimulation. These findings suggest that neural responsiveness, including ECAP measures, may evolve with increasing CI experience.

On the other hand, Hughes et al. (2000) found that ECAP thresholds and amplitudes remained relatively stable over time in adult CI users, particularly in the early months post-activation. This may support our current findings, in that ECAP-derived optimal IPI might remain consistent during the initial months after implantation, especially in adult users with limited auditory plasticity.

Nevertheless, the narrow range of CI experience in our sample limits the ability to detect potential longitudinal effects. It remains possible that longer-term CI use could result in enhanced neural encoding or temporal resolution, which may in turn shift the optimal IPI. Future studies incorporating broader timeframes and longitudinal tracking would help determine whether optimal IPI adapts with extended CI use.

Several studies have explored the relationship between temporal parameters such as Inter-Pulse Interval (IPI) and hearing performance in CI users. For instance, shorter refractory recovery periods—implied by shorter optimal IPIs—have been associated with better temporal resolution and speech perception outcomes (Xu and Pfingst, 2008; Goldwyn et al., 2012). Conversely, longer optimal IPIs may indicate reduced neural health or increased channel interaction, potentially limiting performance. Although our study did not directly assess speech outcomes, the observed relationship between deafness duration and IPI suggests that temporal neural responsiveness could influence downstream auditory processing. Future studies should directly examine how ECAP-based IPI measures relate to behavioral speech perception.

## 4 Conclusion

This study has revealed the existence of personalized optimal IPI for CI users, emphasizing the need for individualized parameter selection in NRM. Age-related effects on IPI selection were evident, with increasing age associated with longer optimal IPIs. Moreover, the duration of deafness played a pivotal role, with longer durations linked to longer optimal IPIs. This research offers nuanced insights into the complexities of auditory neuroscience and underscores the importance of personalized IPI selection in optimizing neural responses in neurostimulation procedures, which can enhance the predictive ability of T- and C-levels using ECAP thresholds for CI users across diverse demographics.

## Data Availability

The raw data supporting the conclusions of this article will be made available by the authors, without undue reservation.
